# Reaction Dynamics in the Chrimson Channelrhodopsin: Observation of Product-State Evolution and Slow Diffusive Protein Motions

**DOI:** 10.1021/acs.jpclett.2c03110

**Published:** 2023-02-06

**Authors:** Ivo H.M. van Stokkum, Yusaku Hontani, Johannes Vierock, Benjamin S. Krause, Peter Hegemann, John T.M. Kennis

**Affiliations:** † Department of Physics and Astronomy and LaserLaB, Faculty of Science, Vrije Universiteit Amsterdam, De Boelelaan 1081, 1081 HVAmsterdam, The Netherlands; ‡ Institut für Biologie, Experimentelle Biophysik, Humboldt-Universität zu Berlin, Invalidenstrasse 42, 10115Berlin, Germany

## Abstract

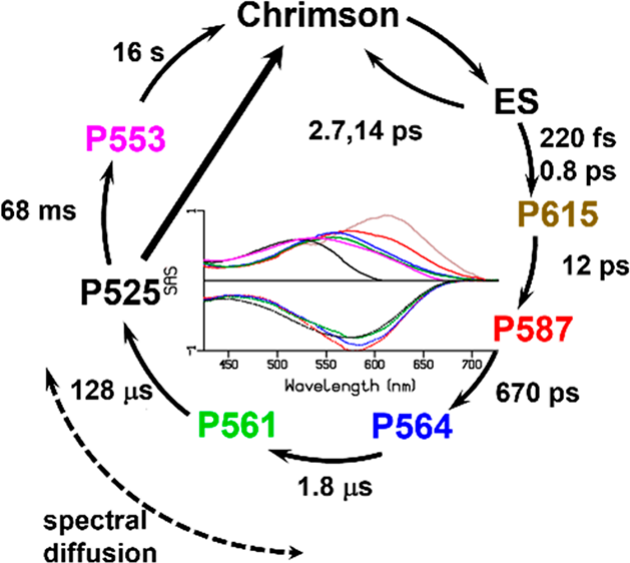

Chrimson is a red-light absorbing channelrhodopsin useful for deep-tissue optogenetics applications. Here, we present the Chrimson reaction dynamics from femtoseconds to seconds, analyzed with target analysis methods to disentangle spectrally and temporally overlapping excited- and product-state dynamics. We found multiple phases ranging from ≈100 fs to ≈20 ps in the excited-state decay, where spectral features overlapping with stimulated emission components were assigned to early dynamics of K-like species on a 10 ps time scale. Selective excitation at the maximum or the blue edge of the absorption spectrum resulted in spectrally distinct but kinetically similar excited-state and product-state species, which gradually became indistinguishable on the μs to 100 μs time scales. Hence, by removing specific protein conformations within an inhomogeneously broadened ensemble, we resolved slow protein backbone and amino acid side-chain motions in the dark that underlie inhomogeneous broadening, demonstrating that the latter represents a dynamic interconversion between protein substates.

Rhodopsins are photoactivatable retinal binding membrane proteins^1,2^ with great impact as modular tools in optogenetics^2,3^ and voltage sensing.^4^ Chrimson is a red-light absorbing channelrhodopsin that passively conducts protons.^5^ It is particularly useful for optogenetics applications^6^ because of the deeper penetration of red light in mammalian tissues^7^ and to be experimentally combined with blue or green absorbing secondary channels or fluorescent sensors (dual-color) applications, e.g., coapplied with the blue anion channel *Gt*ACR2^8^ or the green Ca^2+^ indicator GCaMP.^9,10^ Recently, a great interest in red-shifted microbial rhodopsins has arisen, in particular, with the discoveries of NeoR^11^ and Bestrhodopsins.^12^ At low pH, the Chrimson absorption maximum is located at 582 nm, which renders it the most red-shifted cation conductive microbial channelrhodopsin. The Chrimson three-dimensional structure has been resolved,^13^ adding to existing structural information on channelrhodopsins^14,15^ and giving insights into the nature of the red-shift of its absorption spectrum. Figure 1 shows the Chrimson X-ray structure, along with an enlarged illustration of the active site showing the protonated retinal Schiff base (RSB) and its hydrogen-bond interactions. Thus far, limited information exists on the reaction dynamics of red-absorbing microbial rhodopsins.^11,16^ Here, we present a comprehensive study of femtosecond-to-second dynamics of Chrimson upon green to red excitation conditions, extensively analyzed by global and target analysis methods.

**Figure 1:**
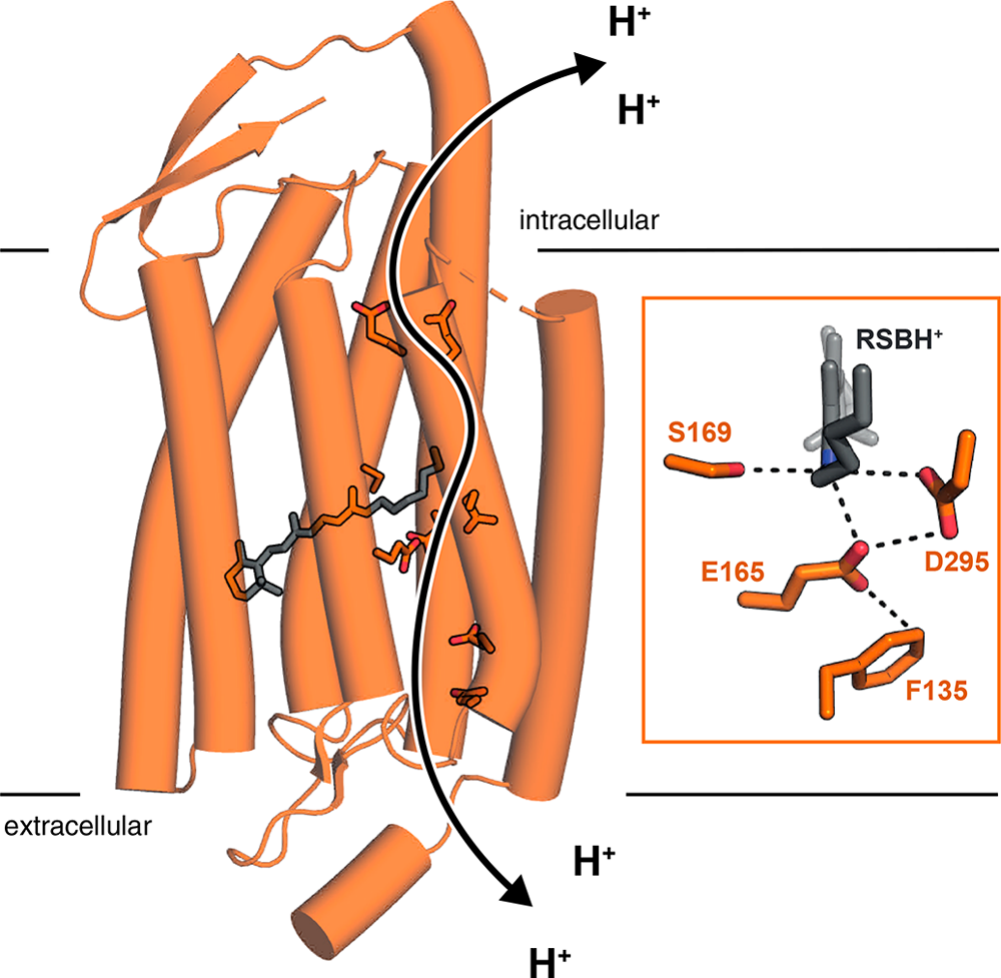
Three-dimensional structure of Chrimson (pdb 5zih) showing the retinal chromophore, pore lining glutamates E1′ to E5′, and the counterion complex. The putative proton pathway is indicated by the black arrow. (inset) Enlarged illustration of the active site with the protonated retinal Schiff base (RSBH^+^) and its direct hydrogen-bond interaction partners.

We carried out femtosecond-to-sub-millisecond (fs-sub-ms) transient absorption (TA) spectroscopy^17^ using a pair of electronically synchronized Ti:sapphire laser systems^18−21^ and μs-to-s flash photolysis spectroscopy on Chrimson at pH 5, at which point it exists in its red-absorbing state. For the TA experiments, two excitation conditions were chosen: 520 nm, which is at the blue edge of the absorption, and 580 nm, which is near the maximum absorption (cf. the minimum in the black dotted curve in Figure 2B). Data taken on the fs-sub-ms TA setup were simultaneously analyzed^22^ with those from flash photolysis, spanning more than 13 decades of time. Nine components S1–S9 were required for an adequate fit of the TA/flash photolysis data for the entire time range, along with a 40 fs component, which we regard as a coherent or cross-phase modulation artifact not considered further. Figure 2A–C and 2D–F show the results in terms of the evolution-associated difference spectra (EADS) with 520 nm and 580 nm excitation, respectively. Figure S1 shows the kinetics along with the result of the global fit, which was considered as excellent. The slight differences between fs-sub-ms TA and flash photolysis are most probably of instrumental origin or may be caused by the continuous measuring light in the case of flash photolysis.

**Figure 2:**
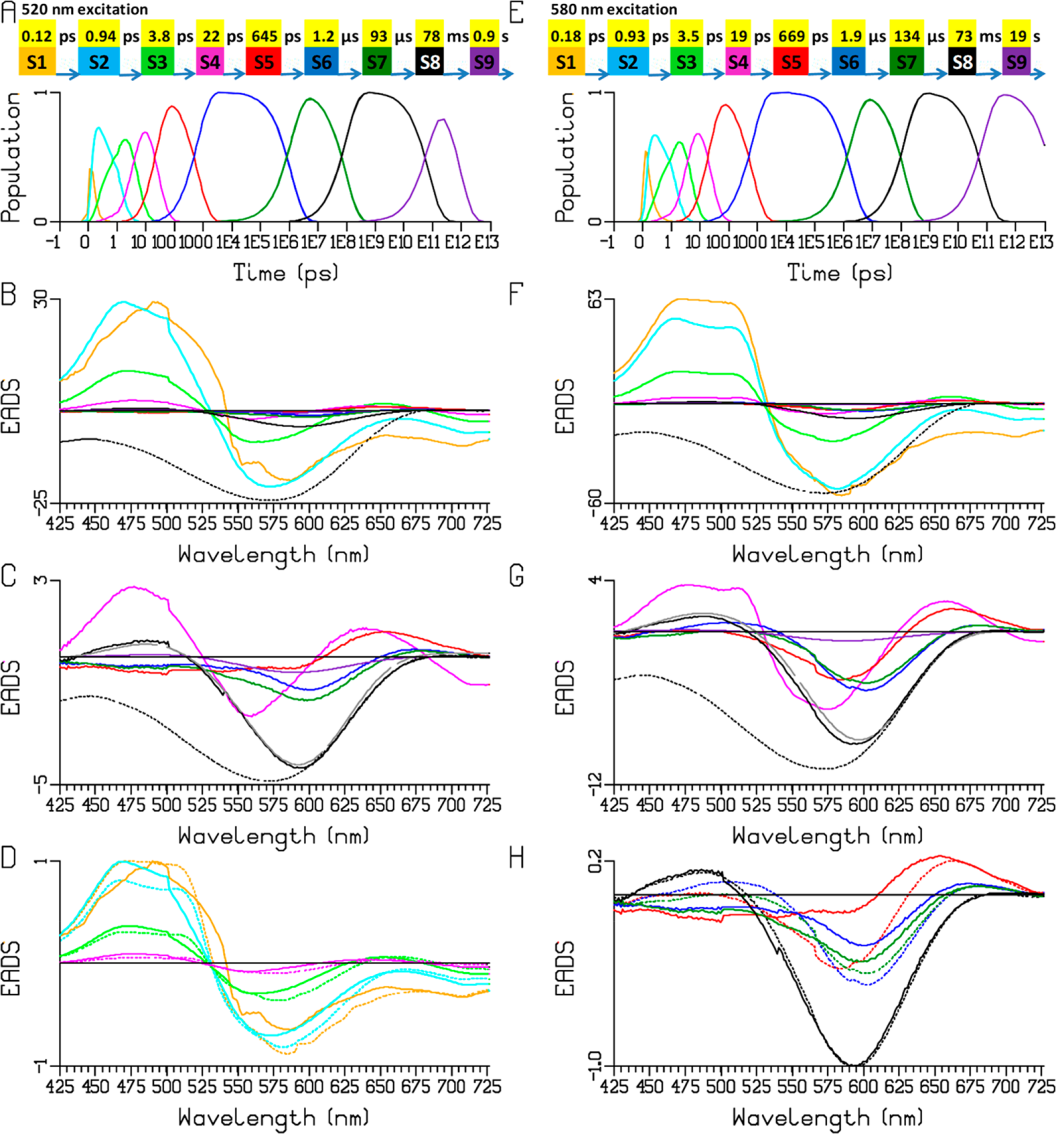
Sequential analysis of the Chrimson reaction dynamics at pH 5.0 upon 520 (A–C) and 580 (E–G) nm excitation. (A, E) Populations of the components, with the kinetic schemes indicated at the top, the lifetimes are in the yellow highlighted cells. Note that the time axis is linear until 1 ps (after the maximum of the instrument response function, IRF) and logarithmic thereafter. (B, C, F, G) EADS (in mOD), in C and G starting from S4. The black dotted curve represents the sign-inverted ground-state absorption spectrum, scaled by 0.2 in C and G. Overlays of scaled EADS of (D) S1–S4, (H) S5–S8, key: 520 (solid) and 580 nm (dotted) excitation.

Immediately after excitation at 520 nm, the first EADS (orange line in Figure 2B, hereafter called S1) represents the excited state with ground-state bleach (GSB, dotted black) around 575 nm along with stimulated emission (SE) at 650–725 nm. Note that the “ground state” refers to the dark adapted state at pH 5.0 maximally absorbing at ∼580 nm. The excited states decay multiexponentially, as SE in combination with excited-state absorption (ESA) around 475 nm are observed in the 0.12 ps (S1, orange), 0.94 ps (S2, cyan), 3.8 ps (S3, light green), and 22 ps (S4, magenta) EADS. Concomitantly, a distinct positive band superimposed on the SE appears at ∼670 nm (cyan), which evolves to 650 nm (light green) in 0.94 ps and to 635 nm (magenta) in 3.8 ps. After the excited states have decayed, a primary photoproduct is observed with a maximum difference absorption around 650 nm (S5, red), which is assigned to a K-like intermediate.^1,19,21,23−27^ S5 evolves in 645 ps to S6 (blue line in Figure 2C), which involves a large decrease of the product absorption near 650 nm and an apparent increase of GSB around 600 nm (blue). S6 then evolves in 1.2 μs to S7 (dark green), involving only minor spectral changes. In 93 μs, the system evolves to S8 (black), which involves a large blue shift of the product difference absorption to 490 nm, concomitant with an apparent increase of the GSB. Finally, from the flash photolysis data, which cover the 1 μs to seconds time scales, and are largely consistent with the data taken on the fs-sub-ms TA setup (black and gray curves in Figure 2C,G), S8 evolves in 78 ms to a much smaller S9 (purple) that decays in 0.9 s. This large drop in the amplitude suggests that S8 partially proceeds to the dark state directly. Strikingly, no M-like intermediate (characterized by near-UV absorption) with deprotonated RSB is observed at any stage of the photocycle, as already observed previously.^16^ The time constants and overall spectral evolution described in Figure 2 are consistent with those reported before at pH 6.^16^ The black dotted curve in Figure 2B,F represents the sign-inverted ground-state absorption spectrum, scaled to envelop the S1 EADS near 600 nm. The large drop in the amplitude of the S8 EADS (black in Figure 2B,F) indicates a much smaller GSB contribution (it is scaled by 0.2 in Figure 2C,G) from which we estimate a quantum yield of ≈20%.

With 580 nm excitation the dynamics (Figure 2E–G) are very similar to those with 520 nm excitation (cf. Figure 2A,E), but specific spectral differences exist between the two excitation conditions. The magenta, red, blue, and dark green EADS clearly differ (cf. the overlays in Figure 2D,H). In particular, the GSB around 500 nm is absent in the 580 nm excitation red, blue, and dark green EADS (dotted vs solid lines). On the other hand, the S8 EADS (Figure 2H, black), which largely represent the terminal transient state, are very similar. The quality of the fit is excellent with both excitation wavelengths (Figure S1). The last intermediate S9, which is populated only sparsely, has a longer lifetime than with 520 nm excitation, of which the origin is unclear.

On fs-to-ps time scales, the excited-state evolution and primary photoproduct formation occur simultaneously, invoking the need for a target analysis to disentangle the various molecular processes.^19,22,28,29^ We will first perform a target analysis for three possible interpretations of the ps evolution in the excited state (Figure 2D). Thereafter we will attempt to interpret the μs to ms differences (Figure 2H). The excited state is characterized by GSB, SE, and ESA (Figure 2D, orange lines). Superimposed on the SE of the excited-state signal, a distinct absorption is evolving in the 625–725 nm spectral region, and the question arises how to interpret these signals. We quantitatively tested three scenarios (**1**–**3**) by means of target models. According to scenario **1**, the positive 625–675 nm band is due to ESA, which would imply that extensive evolution on the excited-state potential energy surface occurs. The kinetic model and species-associated difference spectra (SADS) are presented in Figure S2. Four excited-state compartments ES1–ES4 are assumed, which evolve according to ES1 → ES2 → ES3 → ES4, and each may contribute to the formation of the primary product P1 (which possesses the same shape as S5 in Figure 2). P1 is assumed to irreversibly evolve according to P1 → P2 → P3 → P4 → P5. We compare two extremes: in model (**1a**), only ES1 and ES2 produce the P1 photoproduct with a common rate, and in model (**1b**), all ES are productive with a common rate. In the latter case most of the product is resulting from the later excited states ES3 and ES4. We estimated the SADS in these two extreme scenarios and conclude that the SADS in scenario (**1b**) are less plausible because the ES3 and ES4 SADS contain features of the P1 SADS (red line in Figures S2 and S3), which is most clear with 580 nm excitation (turquoise and maroon lines in Figure S3G). Thus, we prefer scenario (**1a**) over (**1b**).

In the remainder we assume that the product is generated only from ES1 and ES2. In scenario (**2**), the positive 625–675 nm band is assumed to be due to a ground-state intermediate (GSI) of the nonreactive molecules that did not enter the photocycle. Such GSIs have been observed in C1C2 channelrhodopsin,^19^ proteorhodopsin,^30^ and in photoactive yellow protein (PYP).^28,31^ In this target analysis, the SADS of ES2, ES3, and ES4 are assumed to be identical, which is required to constrain the fit, and all excited states decay via a GSI to the dark state. The thus-estimated GSI SADS (brown in Figure S4C,G) do not have a realistic shape, especially with 580 nm excitation, where it resembles the ESA around 475 nm (Figure S4C). Moreover, the GSI lifetime of 7 ps is significantly longer than those determined previously in C1C2 and proteorhodopsin (1.0 and 2.5 ps, respectively). Thus, we discard scenario (**2**).

Finally, we consider scenario (**3**), where the evolution of the positive 625–675 nm band is assumed to result from a primary product state P0 that precedes P1. Figure 3 shows the kinetic scheme (A,E) and the SADS (C,G) for 520 and 580 nm excitations, respectively. We first describe the results with 580 nm excitation. Again, the SADS of ES2, ES3, and ES4 are assumed to be virtually identical, and the product P0 (brown compartment and SADS) is introduced. The SADS of P0 and P1 (red SADS) are assumed to be identical below 525 nm to constrain the fit. The SADS of ES1–ES4 all show SE in the 675–725 nm region, indicating that they indeed all denote excited-state compartments. In addition, they show ESA around 475 nm. We find that P0 formation takes place from the ES1 and ES2 compartments with 0.22 and 0.77 ps lifetimes, with 5% and 18% yields, respectively, resulting in an overall quantum yield for P0 formation of ≈20%. This quantum yield is rather low as compared to that of bacteriorhodopsin (60%)^32^ and C1C2 (30%).^19^ The ES3 and ES4 compartments with lifetimes of 2.7 and 14 ps constitute a small fraction of the overall excited-state population and decay to the ground state without forming a photoproduct. P0 evolves to P1 in 12 ps. Its SADS shows a GSB and product absorption that are spectrally similar to those of P1, suggesting that P1 is a relaxed form of P0. Such evolution is reminiscent of the J to K transition observed in bacteriorhodopsin and other microbial rhodopsins and has been assigned to a vibrational cooling process of the newly formed isomerized product occurring in about 3 ps.^27,33^ The P0 lifetime of 12 ps would be too long for such a process, which implies that a different type of relaxation process may underlie the P0 to P1 evolution. The evolution of the SADS of the products P1, P2, P3, P4, and P5 estimated in the target analysis (Figure 3H) follows what is described above for the sequential analysis (the S5–S9 EADS). We applied the same kinetic model to the data with 520 nm excitation, the results of which are shown in Figure 3B–D. As for the sequential analysis of Figure 2, the fitted rate/time constants were similar to those with 580 nm excitation, but distinct differences arose in the SADS.

**Figure 3:**
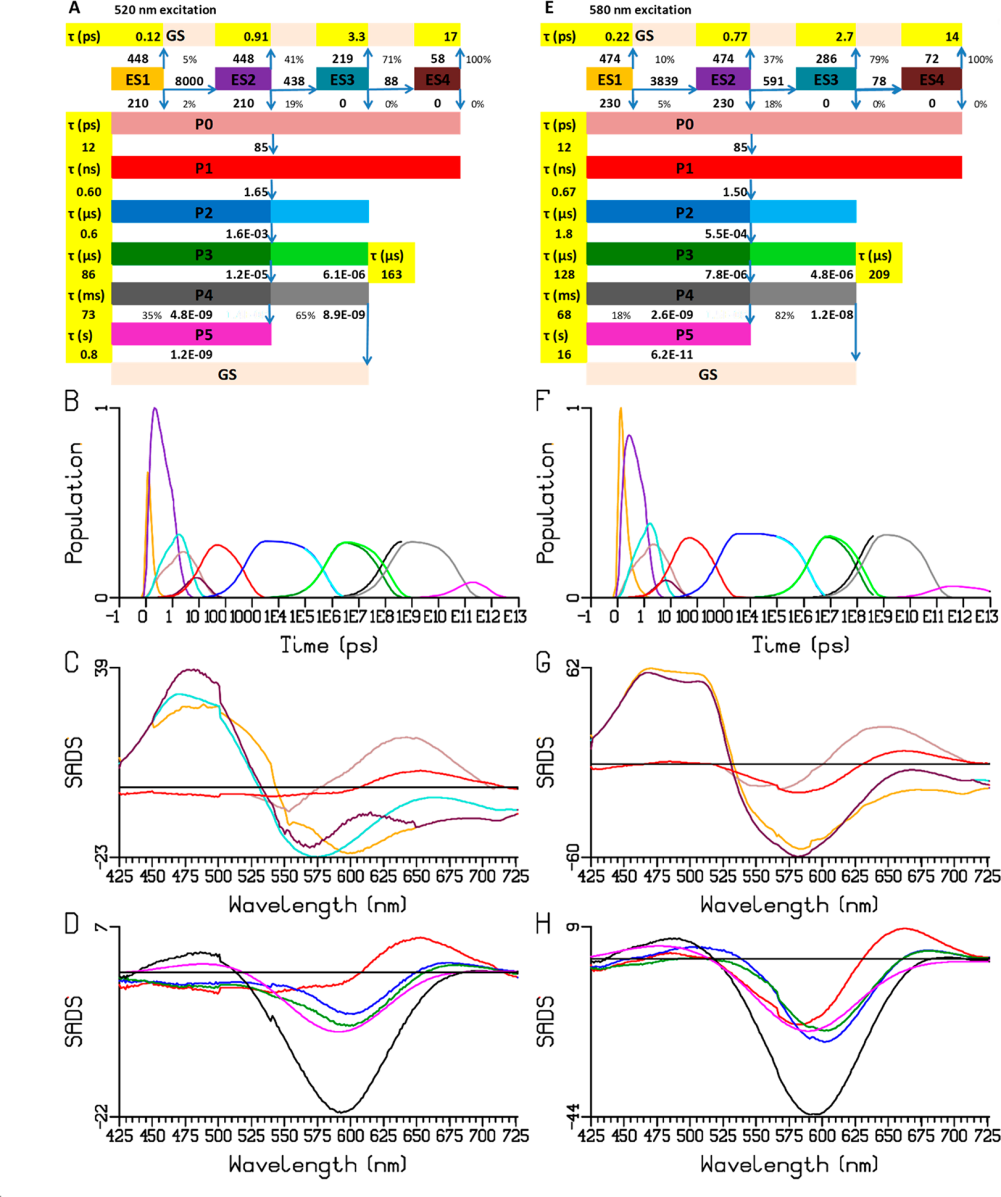
Target analysis of the Chrimson reaction dynamics at pH 5.0 upon 520 (A–D) and 580 (E–H) nm excitation according to the kinetic scheme (**3**) (A, E) in which the SADS of ES2, ES3, and ES4 are assumed to be virtually identical, and a product P0 (brown SADS) is introduced that precedes P1. (B, F) Populations of the species. Note that the time axis is linear until 1 ps (after the maximum of the IRF) and logarithmic thereafter. (C, G) SADS (in mOD) of ES1–ES4, P0, and P1. (D,H) SADS of P1–P5. Key: ES1–ES4: orange, purple, turquoise, maroon; P0–P5: brown, red, blue, dark green, black, magenta. Cyan, green, gray, and magenta in (B, F) refer to flash photolysis populations.

The data are equally well-described with schemes (**1a**), (**1b**), (**2**), and (**3**). However, the implausibility of some of the SADS in Figures S3 and S4 allow us to reject schemes (**1b**) and (**2**). In addition, scheme (**1a**) has more different SADS than scheme (**3**), and together with the reasonable interpretation of P0 as a precursor state to P1, we therefore prefer the latter, although we cannot strictly exclude the possibility that the positive band at 625–675 nm results from extensive evolution on the excited-state potential energy surface.

We now consider the origin of the μs-to-ms spectral differences between the 520 and 580 nm excitation conditions (Figure 2H). Under such conditions, excitation occurs either on the blue edge or at the maximum of the absorption spectrum, respectively. First, we note that, in Chrimson at pH 5, the absorption spectrum is significantly broader than that of bacteriorhodopsin.^34,35^Figure S5 shows the absorption spectra of Chrimson and bacteriorhodopsin,^35^ along with a skewed Gaussian fit. In Chrimson, we find a Gaussian width of 3882 cm^–1^ (full width at half-maximum (fwhm)) and a skewness parameter of 0.488, whereas in bacteriorhodopsin, the numbers are 2897 cm^–1^ and 0.336, indicating that the spectral width is ∼900 cm^–1^ larger in Chrimson. In light-adapted bacteriorhodopsin, the absorption bandwidth is mainly determined by an extensive homogeneous broadening combined with a strong vibronic coupling to intramolecular modes.^34,36,37^ The large homogeneous broadening is a direct consequence of the instantaneous photoinduced charge transfer from the protonated Schiff base toward the β-ionone ring, which induces a dielectric response in the protein matrix^34,38^ and, hence, constitutes a general property of the protonated RSB in rhodopsins (with the notable recent exception of NeoR^11,39^). The inhomogeneous broadening of bacteriorhodopsin has been estimated at only 200–400 cm^–1^,^34,36,37^ thereby contributing only slightly to the absorption bandwidth. The question now arises what the origin is of the broad absorption spectrum of Chrimson, i.e., if the inhomogeneous broadening is significantly larger than that of bacteriorhodopsin or that other causes such as isomeric composition heterogeneity apply.

The ES1–ES4 excited-state SADS with excitation at 520 nm have very similar spectral shapes as those with 580 nm excitation but are overall blue-shifted by 10–20 nm. Also, the primary K-like photoproduct P1 (red lines, Figure 3) is overall blue-shifted at 520 nm excitation with respect to that at 580 nm excitation (Figure 2H). These observations indicate that a subpopulation of blue-shifted Chrimson is selectively excited at 520 nm. A key observation is that, in the evolution from P1–P5, the spectral differences between the SADS gradually decrease and essentially disappear when arriving at P4 at the 100 μs time scale (black lines in Figure 2H). We may readily interpret this result by presuming that Chrimson exhibits extensive inhomogeneous broadening in the ground state: with either 520 or 580 nm excitation, a spectral subset of Chrimson molecules is selected giving rise to the observed spectral differences at early times. Inhomogeneous broadening is often regarded as “static inhomogeneity” caused by conformational substates or disorder of molecular systems: in proteins this may be associated with minor structural differences such as disorder in the polymeric peptide-bond backbone structure, side-chain rotamers, or different hydrogen-bond patterns that may affect the absorption wavelength of the protein-bound chromophore. At physiological temperature, i.e., in the presence of significant thermal energy, slow transitions take place between conformational substates, which washes out the initial spectral differences in the TA signals that were caused by the spectrally selective excitation. The latter process is known as spectral diffusion: it applies to the ensemble of proteins that was not excited and determines the negative GSB signals but may also apply to the ensemble of product states for which the evolution is thermally driven, i.e., from the K-like intermediate onward, and which determine the positive absorption signals. We thus assign the gradual disappearance of the spectral differences on the μs time scale to spectral diffusion; we note that spectral diffusion phenomena on μs-ms time scales have been characterized in proteins by single molecule spectroscopy.^40^ To the best of our knowledge, this is the first time that such spectral diffusion phenomena have been observed and characterized on protein systems using transient absorption spectroscopy.

The observation that the spectral differences vanish on μs time scales excludes the possibility that the spectral broadening of Chrimson is caused by RSB isomeric differences, as equilibrations involving such processes usually take much longer, i.e., time scales of minutes to hours.^41^

We aim to determine the absolute spectra of the transient product states, since it does not become directly clear from the sequential and target analysis of Figures 2 and 3 what their precise absorption properties are, while these are important to assign them to specific intermediate states and relate them to transient states previously reported for microbial rhodopsins. This is especially true for P2–P5, where the respective EADS and SADS are dominated by GSB, which makes it difficult to pinpoint their absolute spectrum. To this end, we modified the target analysis of Figure 3 by using the experimental ground-state absorption spectrum to describe the GSB contribution to the data (cf. the black dotted lines in Figure 2), effectively transforming the Species-Associated Difference Spectra (SADS) into Species-Associated Spectra (SAS). We emphasize that this assumption is an approximation given the significant inhomogeneous broadening in the Chrimson absorption spectrum and the resulting spectral selection effects described above. Nevertheless, we consider it instructive to carry out this exercise with SAS; the results are shown in Figure 4. The color coding is the same as for the target analysis of Figure 3. The amplitude by which the ground-state absorption spectrum is effectively added to the SADS is an important parameter: we estimate the relative contribution of the GSB by assuming that the black SAS (Figure 4C), which has a lifetime of ∼70 ms, is significantly blue-shifted with respect to the ground-state absorption, and is essentially the terminal transient state, has zero absorbance above 610 nm. It is furthermore assumed that, upon formation of P1, no losses occur during the photocycle up to P4, thereby fixing the GSB amplitude throughout the evolution of P1–P4. A similar procedure was employed to quantitatively describe the bacteriorhodopsin photocyle.^35^ The fit quality of this target analysis is excellent with both excitation wavelengths (Figure S6).

**Figure 4:**
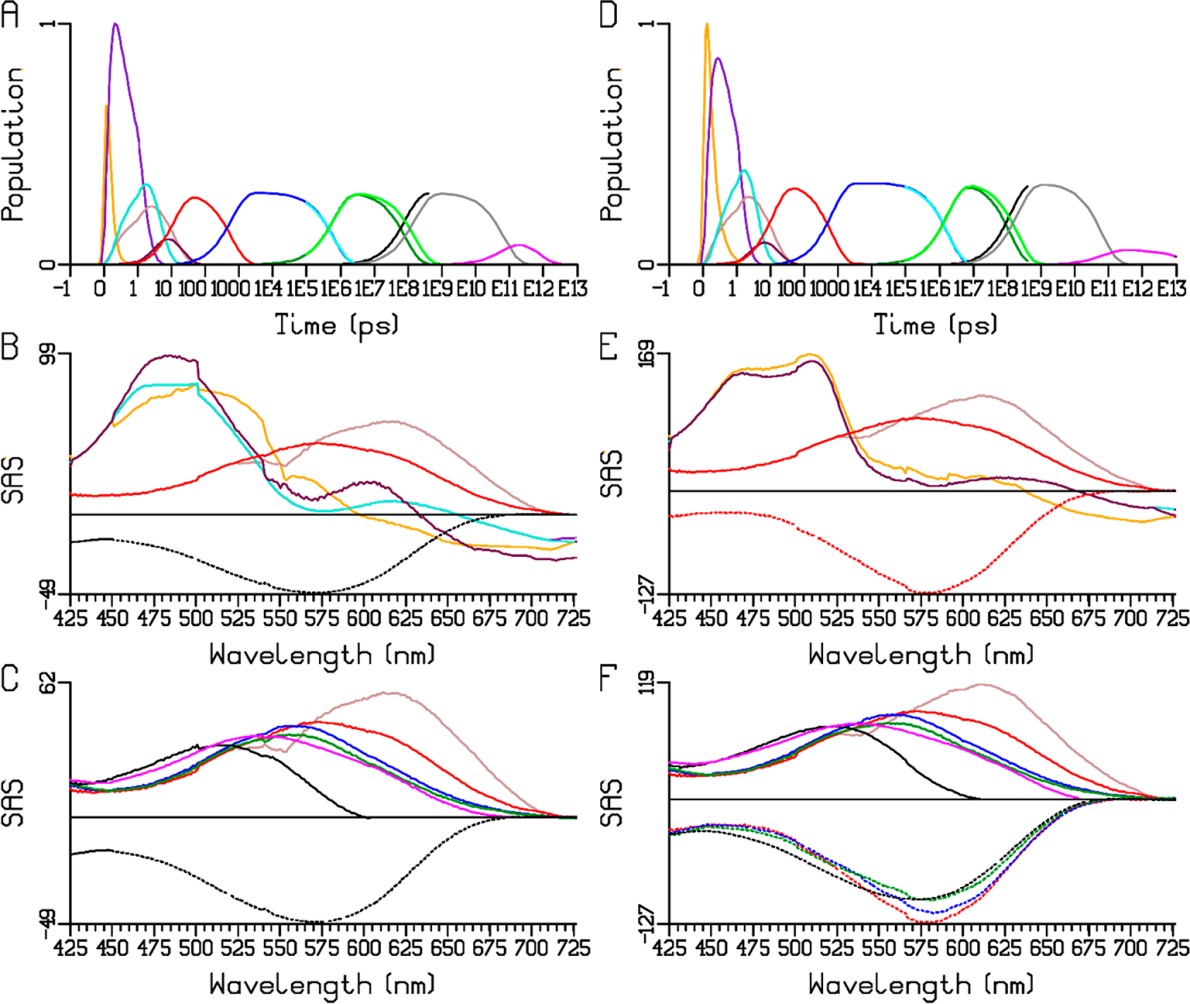
Target analysis including the GSB according to the kinetic scheme (**3**) in Figure 3A,E of the Chrimson reaction dynamics at pH 5.0 upon 520 (A–C) and 580 (D–F) nm excitation. (A, D) Populations of the species. Note that the time axis is linear until 1 ps (after the maximum of the IRF) and logarithmic thereafter. (B, E) GSB (dotted) and SAS (solid, in mOD) of ES1–ES4, P0, and P1. (C, F) GSB (dotted) and SAS (solid) of P0–P5. Key: ES1–ES4: orange, purple, turquoise, maroon; P0–P5: brown, red, blue, dark green, black, magenta. Cyan, green, gray, and magenta in (A, D) refer to flash photolysis populations.

Figure 4B,E shows the SAS of the ES1–ES4, P0, and P1, whereas Figure 4C,F shows the P0–P5 SAS, with, respectively, 520 and 580 nm excitation. The excited-state SAS follow the characteristics described extensively in Figure 3. We note that, in addition to the main ESA around 475 nm, a smaller ESA around 600 nm is present. The P0–P5 SAS have been fitted with skewed Gaussian shapes (Figure S7), resulting in the parameters of Table S2. We henceforth indicate the intermediates by their absorption maximum, with P0 corresponding to P615 and P1 to P587. The P587 SAS (Figure 4, red line) shows a broader absorption edge in the red with respect to the Chrimson absorption spectrum (shown inverted as the modeled GSB with the dotted black line in Figure 4B), indicating that it represents a K-like intermediate. However, it is much broader than that estimated for K intermediates in other microbial rhodopsins,^35^ and its absorption maximum nearly coincides with the Chrimson absorption spectrum. This is a consequence of the use of the Chrimson absorption spectrum to model the GSB: in reality, the actual GSB will be narrower and more blue-shifted because of spectral selection with the 520 nm excitation. The next P2 SAS (Figure 4C,F blue line) evolves from the K-like intermediate in ∼670 ps and has a maximum at 564 nm. We denote this P2 intermediate as P564. It features a long tail to the red from 650–700 nm, which in the EADS of Figure 2C,G becomes apparent as a positive difference absorption, which suggests that P564 may exist in equilibrium with the K-like intermediate on this time scale. P564 evolves to the next P3 SAS in ∼1.8 μs (Figure 4F, blue to dark green evolution), which involves only a minor spectral change and probably involves a slow structural change of a single relaxing intermediate. We denote the dark green P3 SAS as P561. P561 then evolves to the next P4 SAS in ∼128 μs (dark green to black evolution). The black P4 SAS has an absorption maximum at ∼525 nm, and we denote the corresponding intermediate as P525. P525 evolves into the final P5 SAS (magenta) in ∼68 ms, whereby the largest P525 fraction evolves directly to the Chrimson dark state. The final SAS has an absorption maximum at ∼553 nm, and the corresponding intermediate is denoted P553. It has a lifetime much greater than 1 s. Figure 5 shows a schematic view of the Chrimson photocycle with the 580 nm excitation lifetimes.

**Figure 5:**
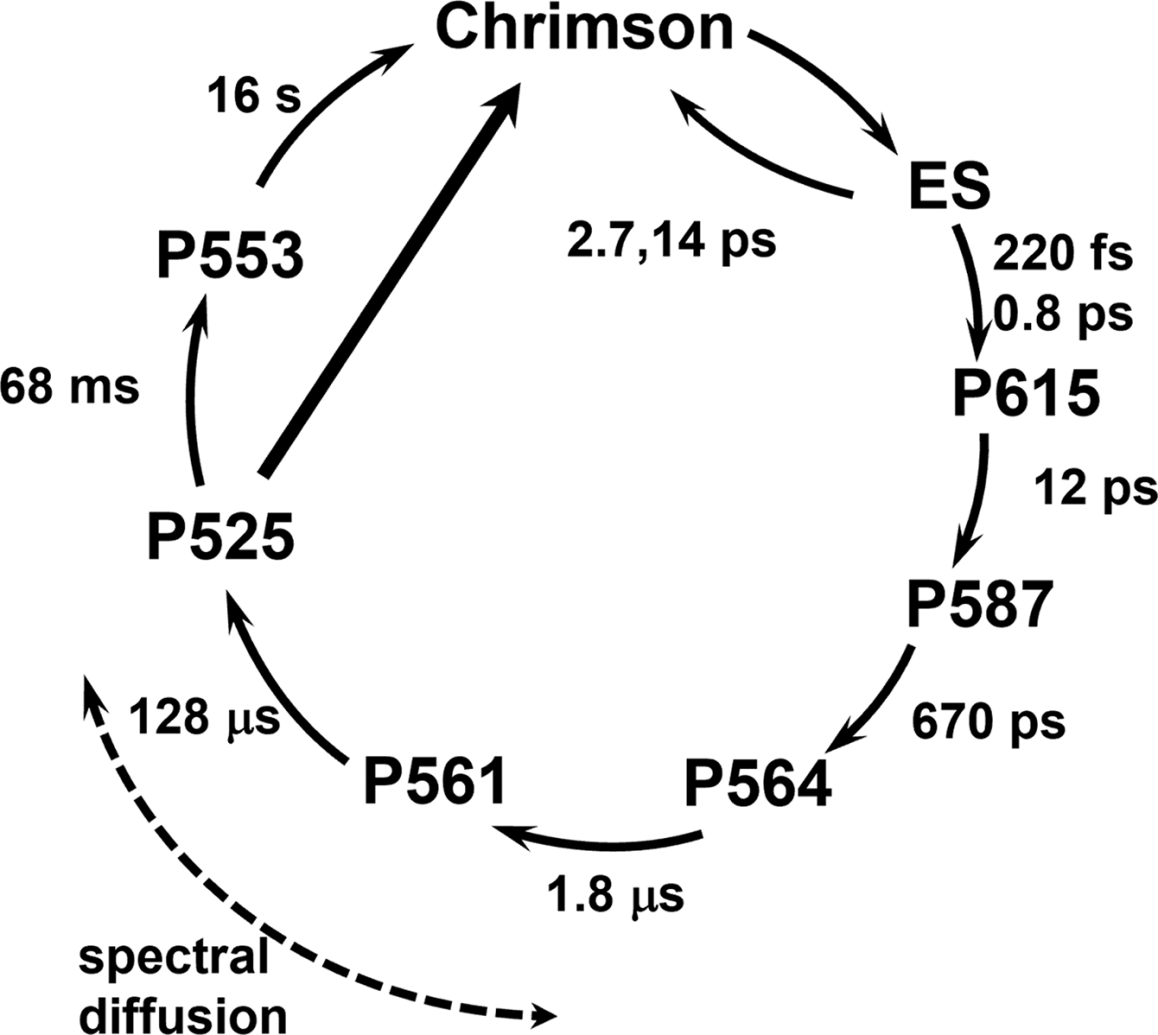
Chrimson photocycle at pH 5, describing the sequential and nonsequential interconversions between spectroscopic intermediates and their lifetimes (with 580 nm excitation), and the occurrence of spectral diffusion phenomena. See text for details.

We now can qualitatively explore the spectral differences between the 520 and 580 nm excited data. In the discussion of Figure 3 above, we interpreted these differences as arising from the effects of inhomogeneous broadening and spectral diffusion. To gain a physical intuition of the underlying dynamics, we applied a simplified model of spectral diffusion to the 580 nm excited data set with the following assumptions.

(1) The same kinetic model is assumed as with 520 nm excitation.
(2) The photoproducts P1–P5 have identical SAS as with 520 nm excitation. We note that this assumption is an approximation because spectral selection will in fact affect these SAS.
(3) Instead of using the Chrimson ground-state absorption as a model for GSB, the GSB of P1–P5 are now estimated by imposing the P1–P5 SAS estimated from the 520 nm excitation target analysis on the data. Thus, in this approach, the effects of spectral selection by the excitation pulse and spectral diffusion are forced to manifest themselves in the GSB of the 580 nm excited data set only.

Figure 4E,F depicts the SAS and GSB estimated from the target analysis with 580 nm excitation. Notably, the GSB of the primary photoproduct P1 (red dotted line) shows a red-shifted maximum and a narrowed spectral profile, consistent with the notion that a spectrally narrow subpopulation of Chrimson has been selected. As time progresses in the P1–P5 evolution, the estimated GSBs shift to the blue slightly and broaden (red to blue to dark green dotted line evolution), and it reaches nearly complete overlap with the steady-state absorption spectrum when P4 is reached in ∼128 μs (black dotted line). Such behavior is consistent with the notion of spectral diffusion, and it suggests that the protein fluctuations that occur on the 100 μs time scale constitute the major driver of the spectral diffusion. The observed time scale is consistent with side-chain rotamer dynamics and peptide bond backbone structural fluctuations. Interestingly, in the Chrimson S169A mutant the absorption spectral bandwidth is significantly smaller.^13^ S169 is located in the retinal binding pocket and hydrogen bonds to the RSB (Figure 1), which suggests that the precise conformation and interaction of amino acid side chains in the retinal binding pocket with the Schiff base modulates the inhomogeneous bandwidth. The spectral diffusion process is unlikely to be associated with hydrogen-bond dynamics mediated by internal waters, as these typically occur on ps time scales. The above analysis of the spectral diffusion phenomena on μs-ms time scales is independent of the kinetic scheme adopted for the product formation on the ps time scale. As evidence thereof, we present the target analysis including the GSB according to the kinetic scheme (**1a**) in Figure S8, cf. the dotted curves in Figure 4F and Figure S8F.

In summary, here we present the femtosecond-to-second photodynamics of the Chrimson channelrhodopsin under two distinct excitation conditions, extensively analyzed by global and target analysis methods. We find that the excited-state dynamics are strongly multiphasic, with fast kinetic components of ∼0.2 and ∼0.8 ps leading to a primary photoproduct, which evolves in ∼12 ps to the isomerized K-like photoproduct and slower components of ∼3 and ∼14 ps that are nonproductive. The quantum yield of the primary photoreaction of Chrimson is lower than in other channelrhodopsins, which might be important for dual-color optogenetic applications of Chrimson, as it increases the range of light intensities where a coexpressed blue-light-sensitive channelrhodopsin can be activated without simultaneously also activating the Chrimson channel. Next, we have identified six photocycle intermediates: P615, P587 (both K-like), P564, P561, P525, and P553. Unlike in other channelrhodopsins,^19,21,23,42,43^ no M-like intermediate with a deprotonated RSB was observed in the Chrimson photocycle.^16^ A striking observation was that the two excitation conditions at 520 and 580 nm produced spectrally distinct excited-state and early product-state spectral signatures, which on time scales of 1–100 μs evolved to become spectrally indistinguishable. We have rationalized this observation with the notions that Chrimson is subject to extensive inhomogeneous broadening, which leads to spectral selection of subpopulations, and that, after excitation, the subpopulations spectroscopically merge through spectral diffusion, caused by thermally driven exchange of protein conformations on time scales of 1–100 μs.

## Supporting Information Available

Detailed methods, eight figures and two tables, detailing the quality of the fit, target analyses using alternative kinetic schemes, spectral fits of the absorption spectra and of the SAS (PDF)
