# Supplementary material for: Reaction Dynamics in the Chrimson Channelrhodopsin: Observation of Product-State Evolution and Slow Diffusive Protein Motions
Source: J Phys Chem Lett. 2023 Feb 6;14(6):1485–93. doi: 10.1021/acs.jpclett.2c03110 (PMC9940203; doi:10.1021/acs.jpclett.2c03110)
Supplement: Supplementary file 1 — jz2c03110_si_001.pdf [file jz2c03110_si_001.pdf]

Supporting Information to

Reaction Dynamics in the Chrimson Channelrhodopsin: Observation of Product-State Evolution and Slow Diffusive Protein Motions

Ivo H.M. van Stokkum[1†], Yusaku Hontani[1,3†], Johannes Vierock[2,4], Benjamin S. Krause[2], Peter Hegemann[2], John T.M. Kennis[1*]

[1]Department of Physics and Astronomy and LaserLaB, Faculty of Science, Vrije Universiteit Amsterdam, De Boelelaan 1081, 1081 HV Amsterdam, The Netherlands.

[2]Institut für Biologie, Experimentelle Biophysik, Humboldt-Universität zu Berlin, Invalidenstrasse 42, 10115 Berlin, Germany

Current addresses:

[3]Brain Research Institute, University of Zurich, 8057 Zurich, Switzerland

[4]Neuroscience Research Center, Charité - Universitätsmedizin Berlin, Charitéplatz 1, 10117 Berlin, Germany

[†]These authors contributed equally.

[*]Corresponding author: email: j.t.m.kennis@vu.nl

Methods

Expression and purification in Pichia pastoris Humanized cDNA of *Cs*Chrimson (GenBank^{TM}: KJ995863.2, 1-345 AA) was inserted in frame with the N-terminal yeast consensus sequence (AATAATGTCT, (Mark Cigan and Donahue 1987)) and a C-terminal TEV protease cleavage site (ENLYFQG) and cloned into pPICZ-C (Thermo Fisher Scientific, Waltham, MA, USA) between *EcoR*I/*Sal*I (Thermo Fisher). Transformation and expression in the methylotrophic yeast *Pichia pastoris* was done as described elsewhere[1, 2] with minor modifications. Proteins were purified by affinity chromatography (His-Trap Crude FF, 5 mL) and desalting (Desalt 16-60). pH was adjusted to pH 7.0 during the desalting step and transferred to pH 5.0 before the measurement.

Transient absorption spectroscopy: Femtosecond to sub-millisecond transient absorption measurements were performed by a pump-probe setup with synchronized 1 kHz Ti:Sapphire amplifiers as reported previously[3]. A 2-mm-thick sapphire plate was used for supercontinuum white light generation, and selected wavelength regions 425 - 725 nm, were detected by a photodiode array. The time delay was varied up to 400 μs at 166 data points with a minimum temporal step of 50 fs. The diameters of the pump and the probe beams at the sample position were ≈200 μm and ≈70 μm, respectively. The wavelength of the pump beam was centered at 520 or 580 nm, and the laser pulse energy was attenuated to ≈400 nJ and ≈300 nJ for 520 nm and 580 nm, respectively. The instrument response function (IRF) was 120 fs full width at half maximum (FWHM), estimated from the global fitting. The flash photolysis experiments were performed as reported previously to observe transient absorption from 1 μs to 10 s[1]. Global and target analysis according to the kinetic schemes in Fig. S3 was carried out as described in.[4] The P1-P4 SAS estimated with 520 nm excitation (Fig.4C) have been used as guidance spectra[5] to estimate the SAS in Fig. 4F. Details of the different kinetic schemes and spectral constraints on the SADS or SAS employed in the target analysis are collated in Table S1.
The absorption spectrum was fitted as a function of the wavenumber with skewed gaussian shapes[4]

$$\varepsilon(\bar{\nu})/\bar{\nu} = \varepsilon_{\max}\exp(-\ln(2)[\ln(1+2b(\bar{\nu}-\bar{\nu}_{\max})/\Delta\bar{\nu})/b]^2)$$

with parameters $\bar{\nu}_{\max}$, $\Delta\bar{\nu}$, and *b* for the location of the maximum, FWHM and skewness.

scheme	Fig.	ES3=ES1	ES3=ES2	ES3=ES4	P0=P1	P4=0	P0=0
sequential	2	-	-	-	-		
(**1a**)	S2,S8	425-450nm	425-525nm	425-540nm			
(**1b**)	S3	425-450nm	425-525nm	425-540nm			
(**2**)	S4	425-450nm	425-725nm	425-725nm			
(**3**)520exc	3,4	425-450nm	425-710nm	425-450nm	425-525nm	600-725nm	710-725nm
(**3**)580exc	3,4	425-450nm	425-725nm	425-715nm	425-525nm	610-725nm	715-725nm

Table S 1. Spectral equality and zero constraints employed in the target analysis with the different kinetic schemes.

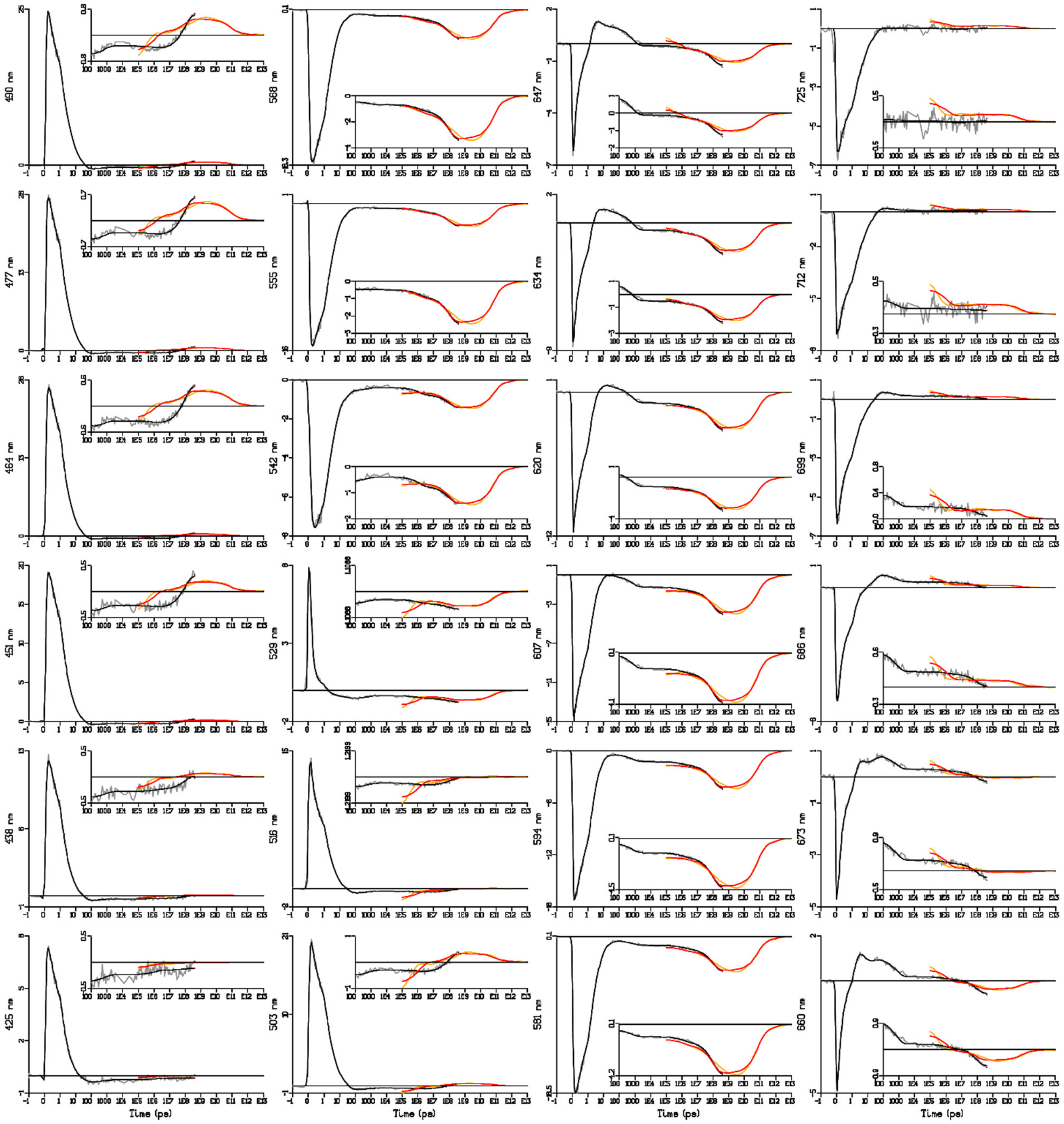

Figure S1A. Selected time traces of Chrimson reaction dynamics at pH 5.0 upon 520 nm excitation. Fs to ms data (in mOD, grey) and global analysis fit (black). Flash photolysis data (orange) and fit (red). Wavelength is indicated in the ordinate label. Note that the time axis is linear until 1 ps (after the maximum of the IRF), and logarithmic thereafter. Insets zoom in on the data after 100 ps.

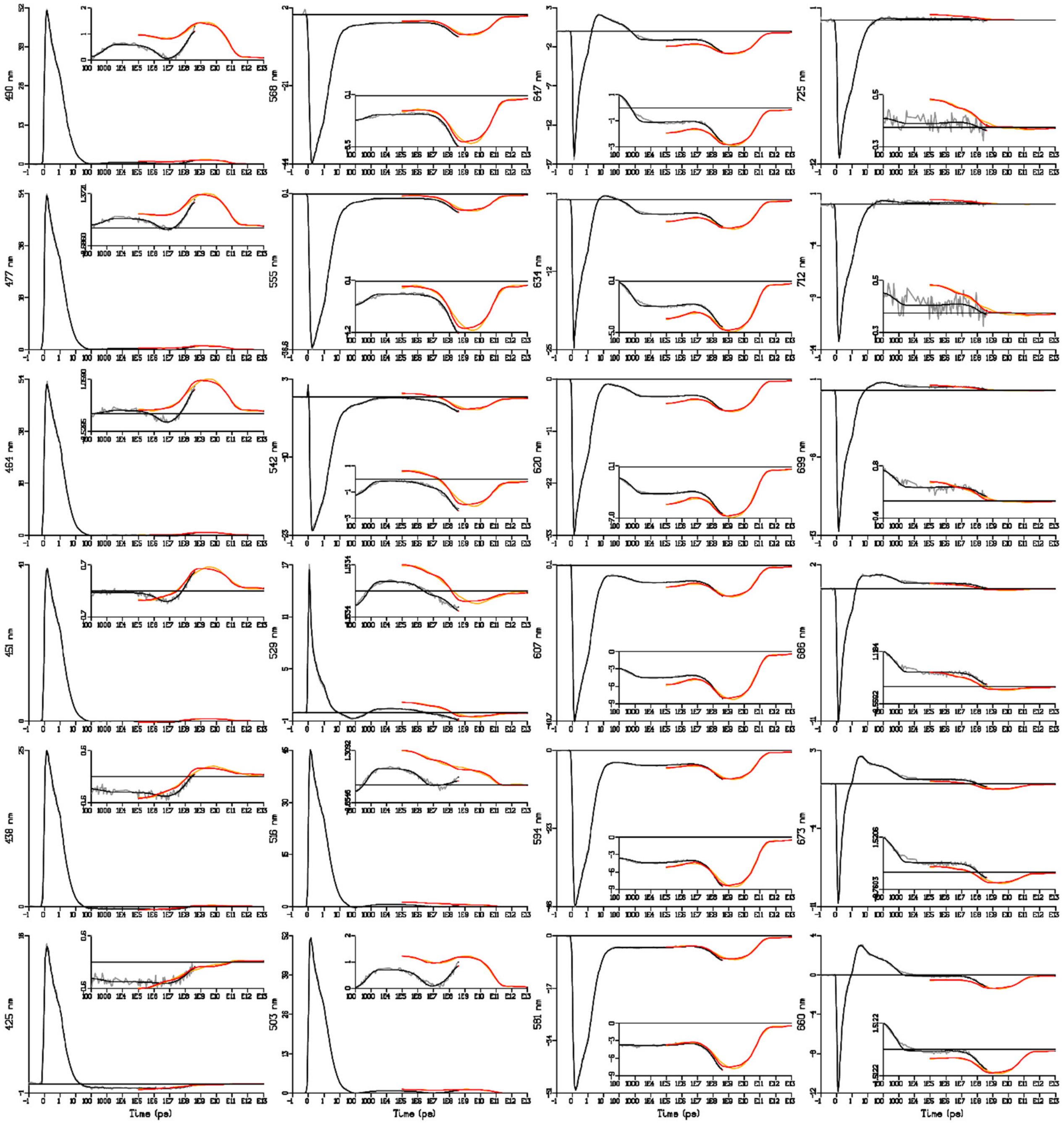

Figure S1B. Selected time traces of Chrimson reaction dynamics at pH 5.0 upon 580 nm excitation. Fs to ms data (in mOD, grey) and global analysis fit (black). Flash photolysis data (orange) and fit (red). Wavelength is indicated in the ordinate label. Note that the time axis is linear until 1 ps (after the maximum of the IRF), and logarithmic thereafter. Insets zoom in on the data after 100 ps.

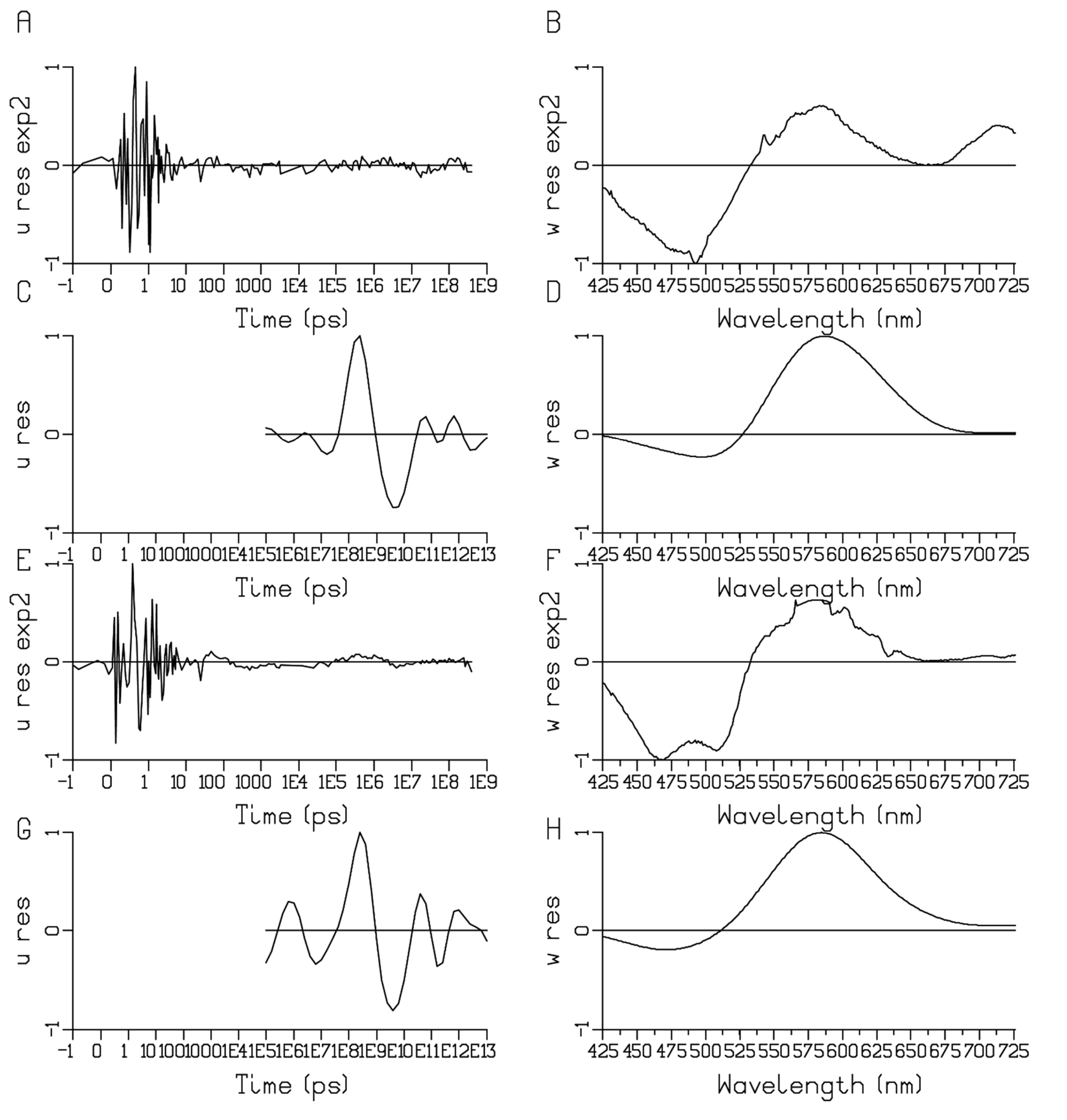

Figure S1C. First left (A,C,E,G) and right (B,D,F,H) singular vectors resulting from the singular value decomposition (SVD) of the residual matrix upon 520 (A-D) or 580 (E-H) nm excitation. Note that the first left singular vector panels (A,E) show no systematic trends, and are dominated by the fluctuations of the laser intensity. Note further that the first left singular vector panels (C,G) do show no systematic trends around 10^8,10^9 ps which are attributed to instrumental differences (e.g., the different set ups, the longer duration of the excitation pulse and the larger amount of measurement light).

Key A,B: 520 nm excitation TR1 (fs-sub-ms TA setup); C,D: 520 nm excitation Time Range 2 (TR2, flash photolysis setup); E,F: 580 nm excitation TR1; G,H: 580 nm excitation TR2. Note that the time axis in (A,C,E,G) is linear until 1 ps (after the maximum of the IRF) and logarithmic thereafter.

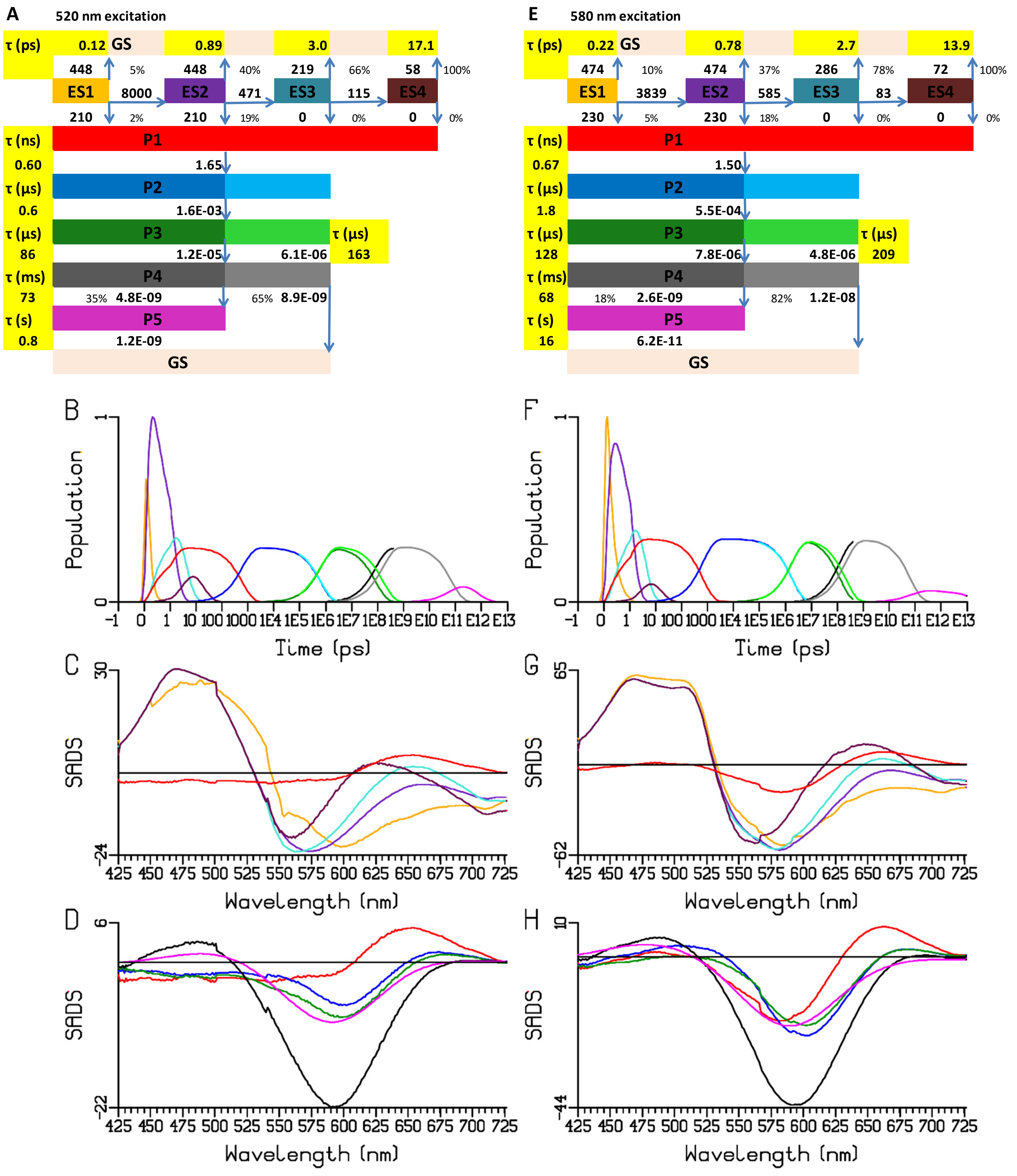

Figure S2. Target analysis (**1a**) of the Chrimson reaction dynamics at pH 5.0 upon 520 (B-D) or 580 (F-H) nm excitation using the kinetic schemes of (A,E) where excited states ES1 and ES2 are productive. In the detailed kinetic scheme (A,E) all rate constants are in ns^{-1}, the lifetimes are in yellow highlighted cells, and the thin percentages indicate the branching. With P2-P5 the lighter colors apply to the flash photolysis data. (B,F) Concentration profiles of ES1-ES4 and P1-P5 upon 520 (B) or 580 (F) nm excitation, using the same colors as in (A,E). (C,G) SADS of ES1-ES4 and P1. (D,H) SADS of P1-P5. Key: ES1 – ES4: orange, purple, turquoise, maroon; P1 – P5: red, blue, dark green, black, magenta. Cyan, green, grey and magenta in (B,F) refer to flash photolysis populations. Note that in the bleach region around 550 nm the SADS of ES2,3,4 in (C,G) are similar.

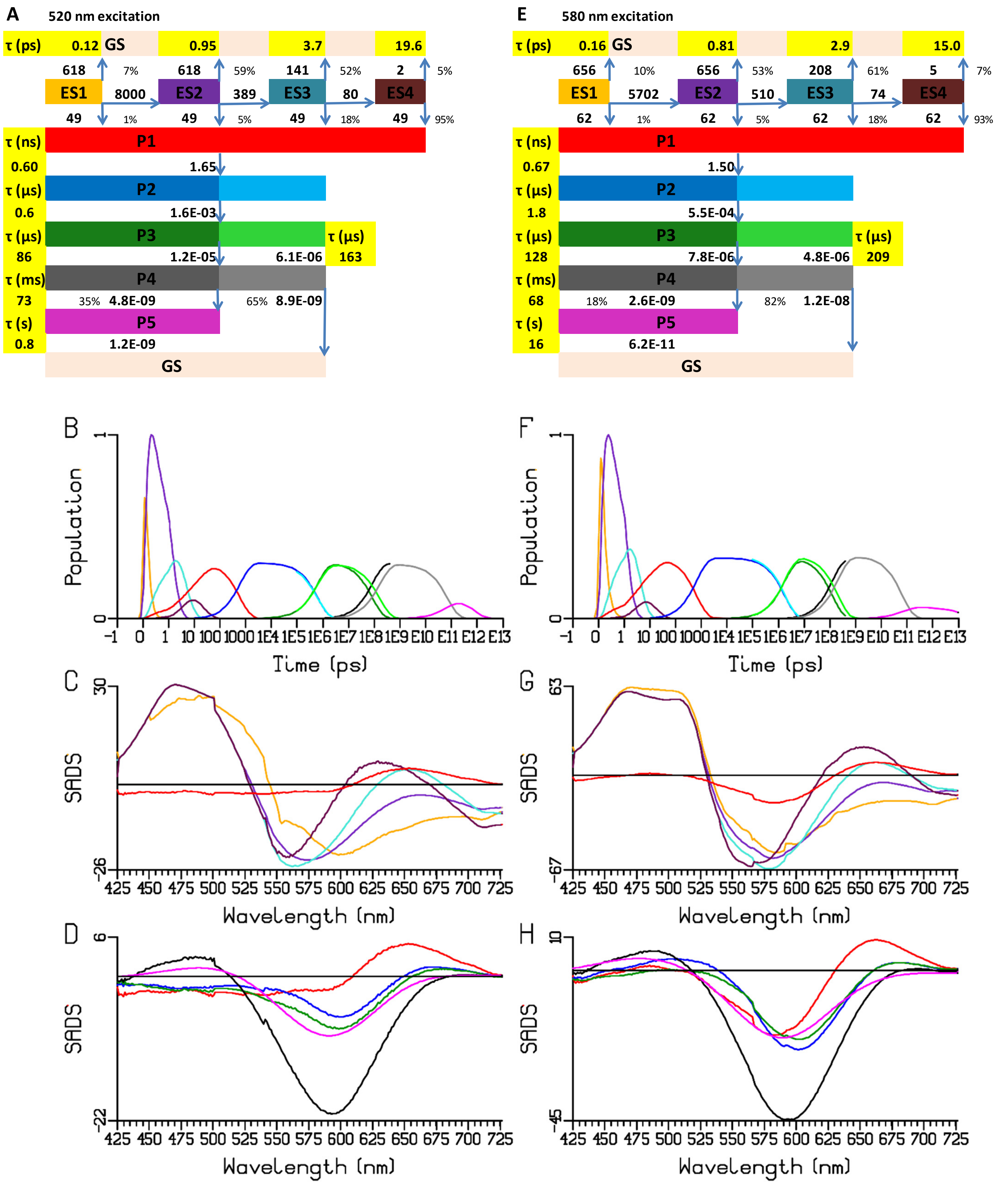

Figure S3. Target analysis (**1b**) of the Chrimson reaction dynamics at pH 5.0 upon 520 (B-D) or 580 (F-H) nm excitation, using the kinetic schemes of (A,E) where all excited states are productive*, with a common rate.* All rate constants are in ns^{-1}, the lifetimes are in yellow highlighted cells, and the thin percentages indicate the branching. With P2-P5 the lighter colors apply to the flash photolysis data. Concentration profiles of ES1-ES4 and P1-P5 upon 520 (B) or 580 (F) nm excitation, using the same colors as in (A,E). (C,G) SADS of ES1-ES4 and P1. (D,H) SADS of P1-P5. Note that the SADS of ES2,3,4 in (G) contain features of P1, well visible in the bleach region around 550 nm. Therefore, this kinetic scheme (**1b**) where all excited states are productive*, with a common rate, is discarded.*

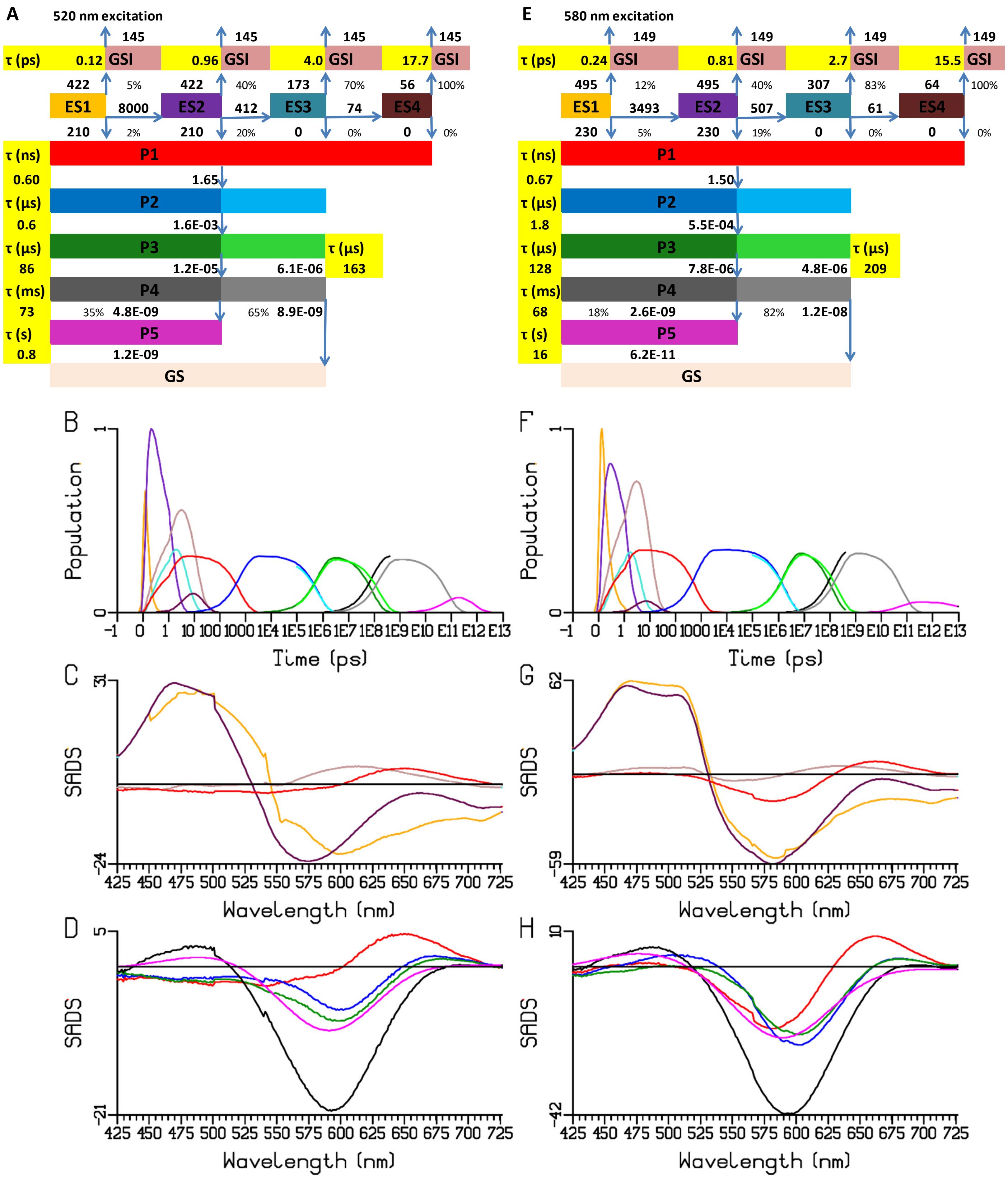

Figure S4. *Target analysis (**2**) of the Chrimson reaction dynamics at pH 5.0 upon 520 (A-C) and 580 (D-F) nm excitation according to scheme (A,E) in which the SADS of ES2, ES3 and ES4 are assumed to be identical, and all excited states decay via a ground state intermediate (GSI, brown SADS). (B,F) Populations of the species. (C,G) SADS (in mOD) of ES1 – ES4 and P1; (D,H) SADS of P1 – P5. Key: ES1 – ES4: orange, purple, turquoise, maroon; P1 – P5: red, blue, dark green, black, magenta. Cyan, green, grey and magenta in (B,F) refer to flash photolysis populations. The estimated GSI SADS (brown in C,G) are not realistic, especially with 580 nm excitation (G) where it shows ESA around 475 nm. Therefore, this kinetic scheme (**2**) where the differences around 640 nm between the ES2, ES3 and ES4 SADS in Fig.S2 are attributed to a GSI is discarded.*

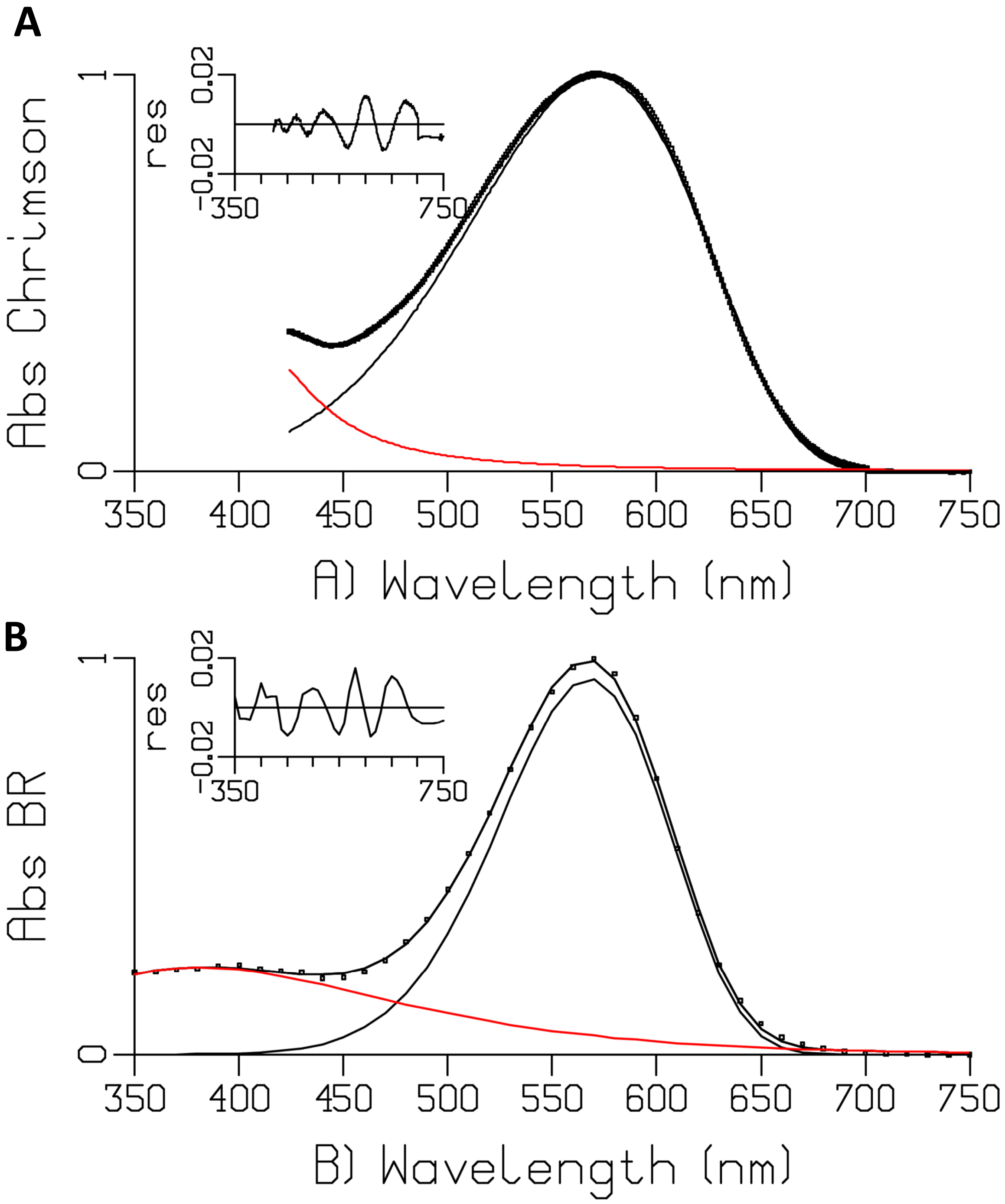

Figure S5. Skewed gaussian fits of the absorption spectrum of Chrimson (A) and bacteriorhodopsin (B). Each spectrum was fitted by a sum of two skewed gaussian bands. The parameters of the main band (black) are (A) $\bar{\nu}_{\max} = 17325$ cm^{-1}, $\Delta\bar{\nu} = 3882$ cm^{-1}, $b = 0.488$ and (B) $\bar{\nu}_{\max} = 17523$ cm^{-1}, $\Delta\bar{\nu} = 2897$ cm^{-1}, $b = 0.336$. A minor band (red) was needed to fit the small absorption at higher energy. Insets depict the residuals which are attributed to vibrational fine structure.

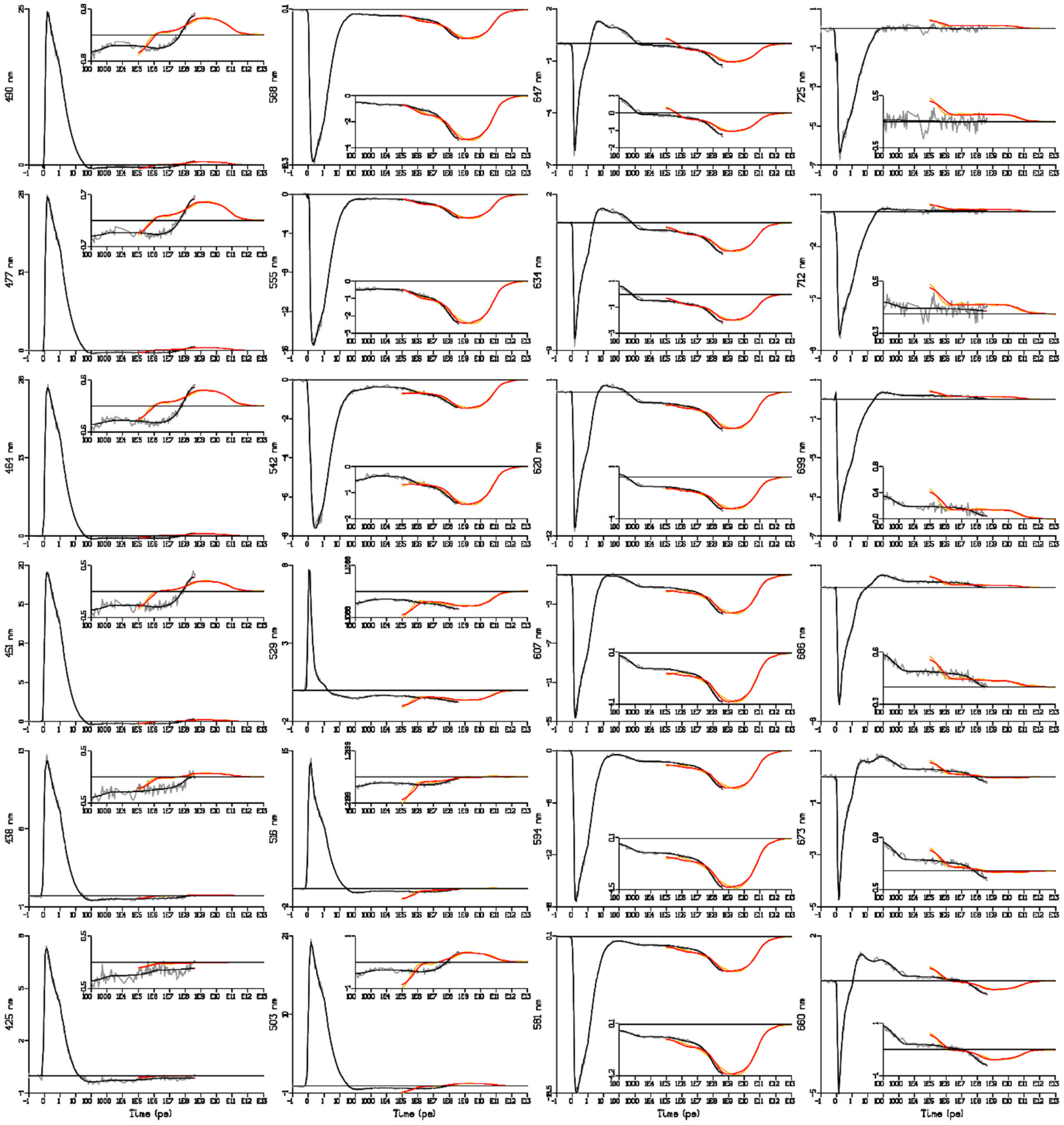

Figure S6A. Selected time traces of Chrimson reaction dynamics at pH 5.0 upon 520 nm excitation. Fs to ms data (in mOD, grey) and target analysis (**3**) fit (black). Flash photolysis data (orange) and fit (red). Wavelength is indicated in the ordinate label. Note that the time axis is linear until 1 ps (after the maximum of the IRF), and logarithmic thereafter. Insets zoom in on the data after 100 ps. Note that in the target analysis slightly different kinetic parameters have been estimated for the flash photolysis data, to describe instrumental differences (e.g., the different set ups, the longer duration of the excitation pulse and the larger amount of measurement light).

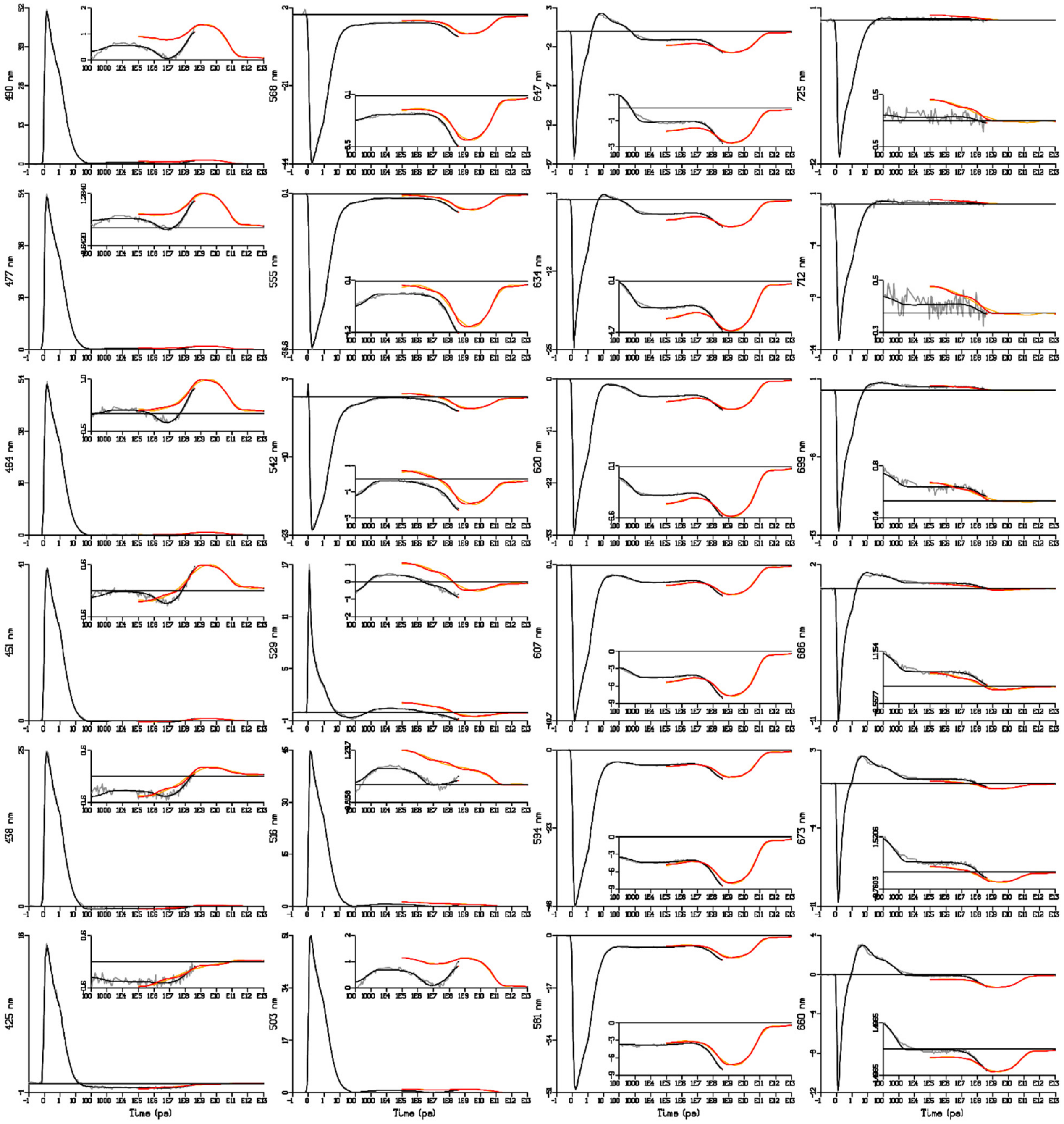

Figure S6B. Selected time traces of Chrimson reaction dynamics at pH 5.0 upon 580 nm excitation. Fs to ms data (in mOD, grey) and target analysis (**3**) fit (black). Flash photolysis data (orange) and fit (red). Wavelength is indicated in the ordinate label. Note that the time axis is linear until 1 ps (after the maximum of the IRF), and logarithmic thereafter. Insets zoom in on the data after 100 ps. Note that in the target analysis slightly different kinetic parameters have been estimated for the flash photolysis data, to describe instrumental differences (e.g., the different set ups, the longer duration of the excitation pulse and the larger amount of measurement light).

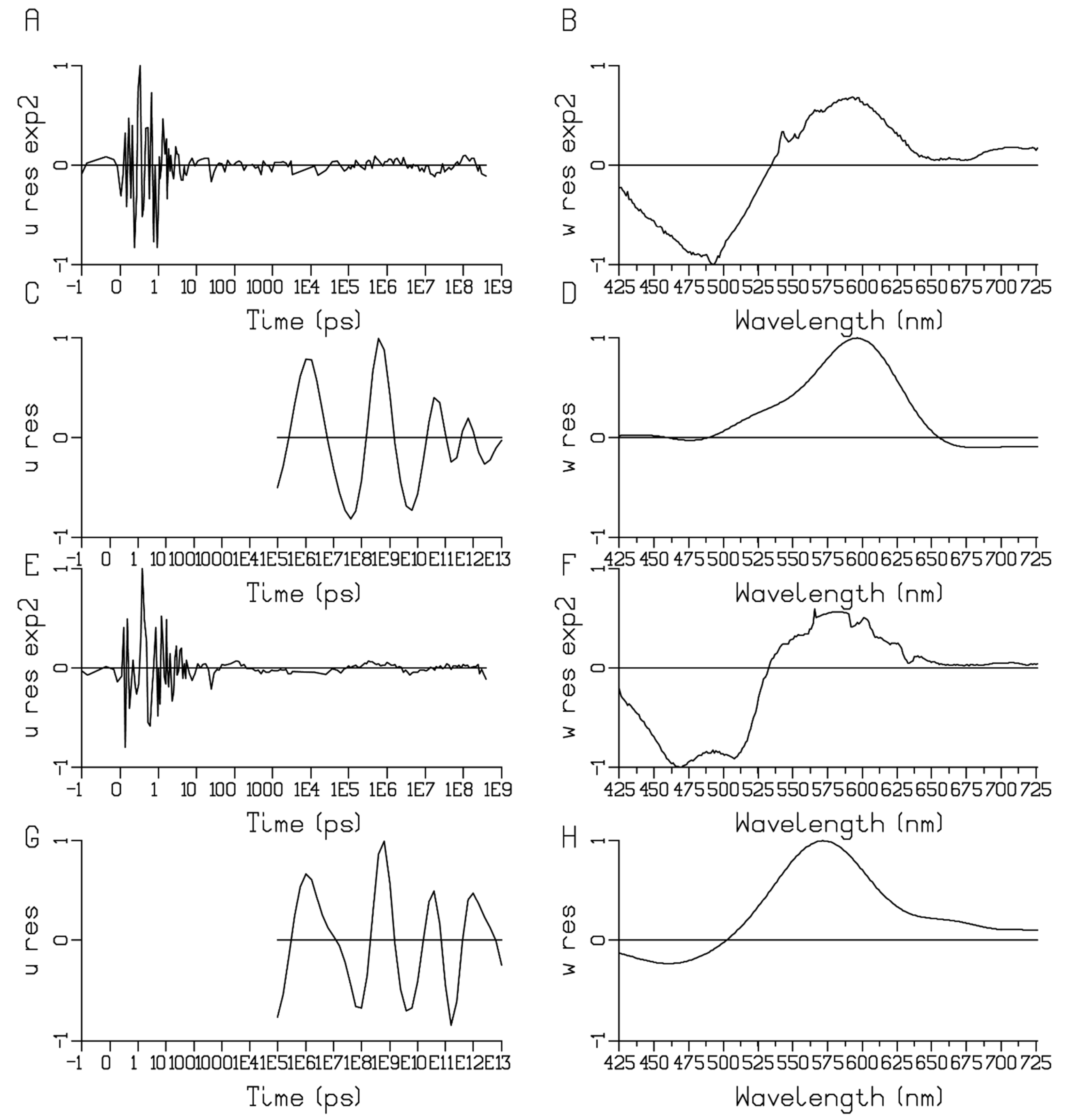

Figure S6C. First left (A,C,E,G) and right (B,D,F,H) singular vectors resulting from the singular value decomposition (SVD) of the residual matrix upon 520 (A-D) or 580 (E-H) nm excitation. Note that all first left singular vector panels (A,C,E,G) show no systematic trends, and are dominated by the fluctuations of the laser intensity. Key A,B: 520 nm excitation TR1 (fs-sub-ms TA setup); C,D: 520 nm excitation Time Range 2 (TR2, flash photolysis setup); E,F: 580 nm excitation TR1; G,H: 580 nm excitation TR2. Note that the time axis in (A,C,E,G) is linear until 1 ps (after the maximum of the IRF) and logarithmic thereafter.

A

1/cm	P0	P1	P2	P3	P4	P5
520 nm exc	16115	17040	17727	17826	19145	18087
580 nm exc	16284	17048	17732	17831	18998	18088

B

nm	P0	P1	P2	P3	P4	P5
520 nm exc	621	587	564	561	522	553
580 nm exc	614	587	564	561	526	553

Table S 2. Location of the maximum absorption $\bar{\nu}_{max}$ in cm^{-1} (A) or λ_{max} in nm (B) estimated from the SAS in Fig.S7.

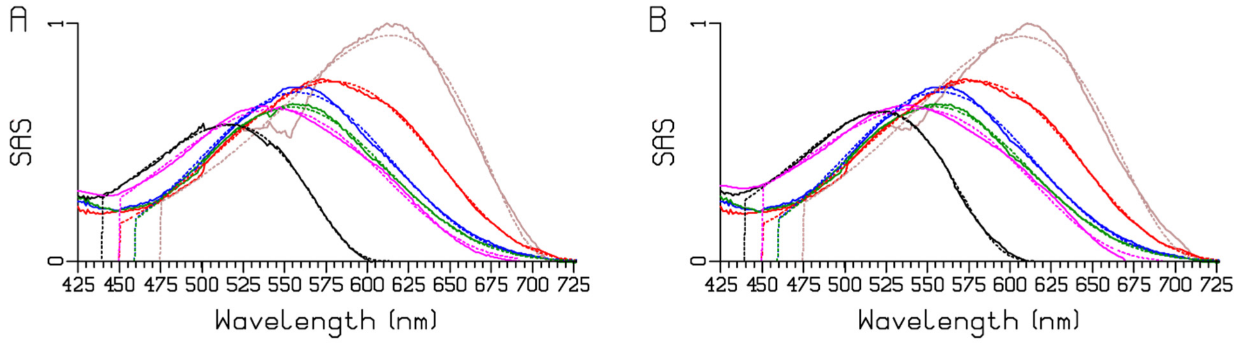

Figure *S7*. SAS of Chrimson P0-P5 upon 520 (A) and 580 (B) nm excitation with superimposed spectral fits (dotted lines) using skewed Gaussian shapes. Key: P0 – P5: brown, red, blue, dark green, black, magenta. The estimated locations of the maximum absorption $\bar{\nu}_{max}$ are collated in Table S 2.

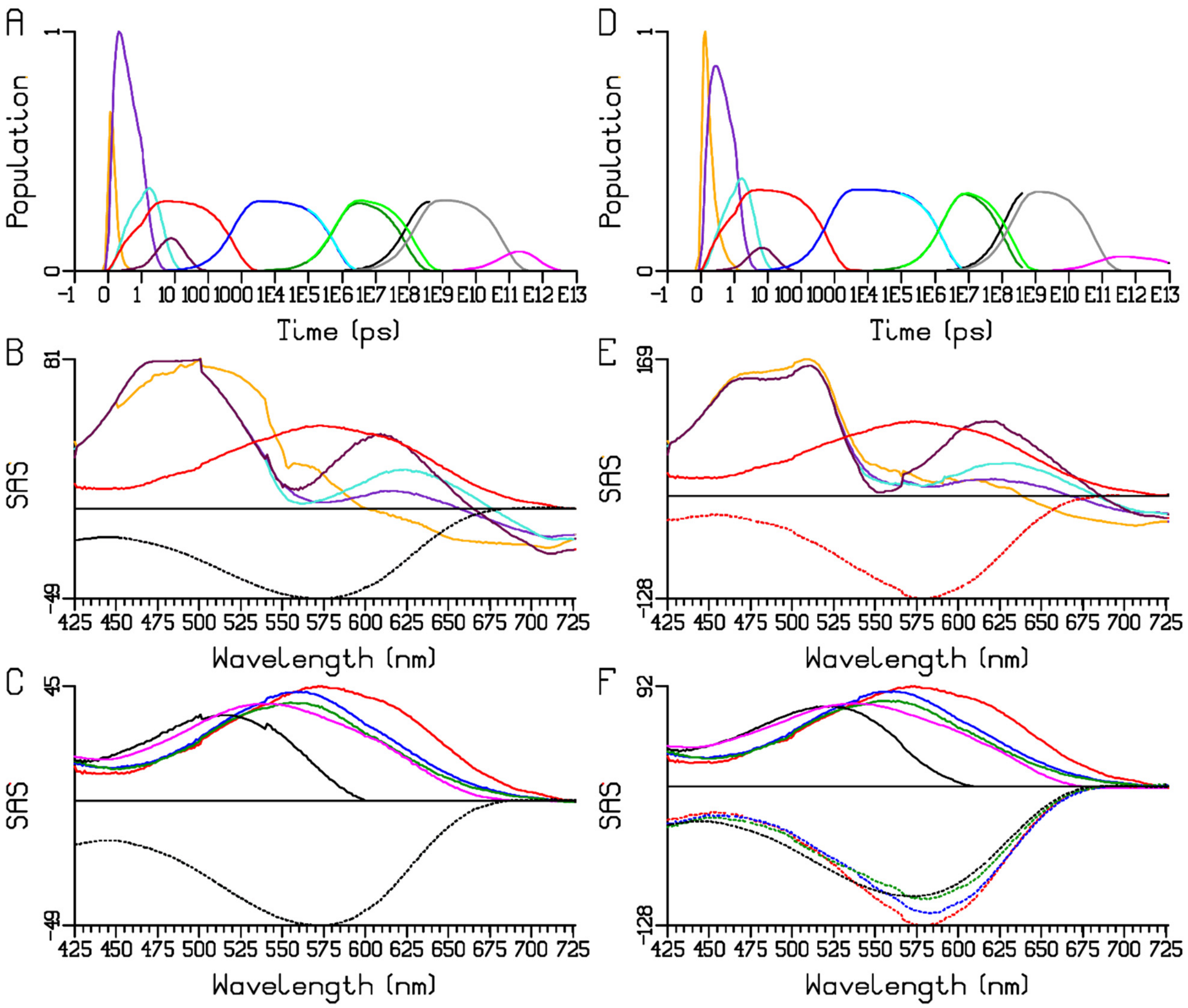

Figure *S8*. Target analysis of the Chrimson reaction dynamics at pH 5.0 upon 520 (A-C) and 580 (D-F) nm excitation, including the GSB according to the kinetic scheme (**1a**) in Fig.S2A,E. (A,D) Populations of the species. Note that the time axis is linear until 1 ps (after the maximum of the IRF), and logarithmic thereafter. (B,E) GSB (dotted) and SAS (solid, in mOD) of ES1 – ES4, and P1 (C,F) GSB (dotted) and SAS (solid) of P1 – P5. Key: ES1 – ES4: orange, purple, turquoise, maroon; P1 – P5: brown, red, blue, dark green, black, magenta. Cyan, green, grey and magenta in (A,D) refer to flash photolysis populations.

References

(1) Luck, M.; Mathes, T.; Bruun, S.; Fudim, R.; Hagedorn, R.; Tra, M. T. N.; Kateriya, S.; Kennis, J. T. M.; Hildebrandt, P.; Hegemann, P. A Photochromic Histidine Kinase Rhodopsin (HKR1) That Is Bimodally Switched by Ultraviolet and Blue Light. *Journal of Biological Chemistry* **2012**, *287* (47). DOI: 10.1074/jbc.M112.401604.

(2) Bruun, S.; Naumann, H.; Kuhlmann, U.; Schulz, C.; Stehfest, K.; Hegemann, P.; Hildebrandt, P. The chromophore structure of the long-lived intermediate of the C128T channelrhodopsin-2 variant. *Febs Letters* **2011**, *585* (24), 3998-4001. DOI: 10.1016/j.febslet.2011.11.007. Bamann, C.; Kirsch, T.; Nagel, G.; Bamberg, E. Spectral characteristics of the photocycle of channelrhodopsin-2 and its implication for channel function. *Journal of Molecular Biology* **2008**, *375* (3), 686-694. DOI: 10.1016/j.jmb.2007.10.072.

(3) Ravensbergen, J.; Abdi, F. F.; van Santen, J. H.; Frese, R. N.; Dam, B.; van de Krol, R.; Kennis, J. T. M. Unraveling the Carrier Dynamics of BiVO4: A Femtosecond to Microsecond Transient Absorption Study. *The Journal of Physical Chemistry C* **2014**, *118* (48), 27793-27800. DOI: 10.1021/jp509930s. Mathes, T.; Ravensbergen, J.; Kloz, M.; Gleichmann, T.; Gallagher, K. D.; Woitowich, N. C.; St Peter, R.; Kovaleva, S. E.; Stojkovic, E. A.; Kennis, J. T. M. Femto-to Microsecond Photodynarnics of an Unusual Bacteriophytochrome. *Journal of Physical Chemistry Letters* **2015**, *6* (2), 239-243. DOI: 10.1021/jz502408n. Mathes, T.; Heilmann, M.; Pandit, A.; Zhu, J. Y.; Ravensbergen, J.; Kloz, M.; Fu, Y. A.; Smith, B. O.; Christie, J. M.; Jenkins, G. I.; et al. Proton-Coupled Electron Transfer Constitutes the Photoactivation Mechanism of the Plant Photoreceptor UVR8. *Journal of the American Chemical Society* **2015**, *137* (25), 8113-8120. DOI: 10.1021/jacs.5b01177. Hontani, Y.; Marazzi, M.; Stehfest, K.; Mathes, T.; van Stokkum, I. H. M.; Elstner, M.; Hegemann, P.; Kennis, J. T. M. Reaction dynamics of the chimeric channelrhodopsin C1C2. *Sci Rep* **2017**, *7* (1), 7217. DOI: 10.1038/s41598-017-07363-w. Hontani, Y.; Broser, M.; Luck, M.; Weissenborn, J.; Kloz, M.; Hegemann, P.; Kennis, J. T. M. Dual Photoisomerization on Distinct Potential Energy Surfaces in a UV-Absorbing Rhodopsin. *Journal of the American Chemical Society* **2020**, *142* (26), 11464-11473. DOI: 10.1021/jacs.0c03229.

(4) van Stokkum, I. H. M.; Larsen, D. S.; van Grondelle, R. Global and target analysis of time-resolved spectra. *Biochimica Et Biophysica Acta-Bioenergetics* **2004**, *1657* (2-3), 82-104. DOI: 10.1016/j.bbabio.2004.04.011.

(5) van Stokkum, I. H. M.; Wohlmuth, C.; Würthner, F.; Williams, R. M. Energy transfer in supramolecular calix[4]arene—Perylene bisimide dye light harvesting building blocks: Resolving loss processes with simultaneous target analysis. *Journal of Photochemistry and Photobiology* **2022**, *12*, 100154. DOI: https://doi.org/10.1016/j.jpap.2022.100154.
